# First record of a mixed species association between Hatinh langur (*Trachypithecus hatinhensis*) and red‐shanked douc (*Pygathrix nemaeus*)

**DOI:** 10.1002/ece3.70122

**Published:** 2024-08-01

**Authors:** Anh Tuan Nguyen, Minh Le, Andrew Tilker

**Affiliations:** ^1^ Faculty of Environmental Sciences University of Science, Vietnam National University Hanoi Vietnam; ^2^ Central Institute for Natural Resources and Environmental Studies Vietnam National University Hanoi Vietnam; ^3^ Re:Wild Austin Texas USA; ^4^ Leibniz Institute for Zoo and Wildlife Research Berlin Germany

**Keywords:** annamites, camera trap, primates, Quang Binh Province, Quang Ninh Protection Forest

## Abstract

In primates, mixed species associations are not common occurrences, and have been linked to both ecological and anthropogenic factors. We present camera‐trapping records of a mixed association between two primates, the Hatinh langur (*Trachypithecus hatinhensis*) and red‐shanked douc (*Pygathrix nemaeus*) and discuss possible hypotheses for this occurrence. To our knowledge, this is the first evidence of such an association in the wild of these two threatened primates, and thus contributes to our limited ecological knowledge of the species.

## Graphical abstract

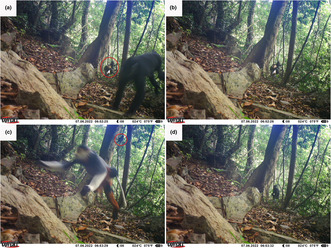

## 1 INTRODUCTION

Mixed species associations have been documented in a range of taxa (Goodale et al., 2020). Most studies have suggested that mixed species associations provide functional benefits to the species involved, including more efficient foraging and enhanced predator avoidance (Haugaasen & Peres, 2009; Terborgh, 1990). Foraging advantages may include reduced effort to find food resources, or increased access to otherwise unavailable food sources (Heymann & Buchanan‐Smith, 2000; Pinheiro et al., 2011; Porter, 2001). Advantages of predator avoidance for a mixed species association may include more efficient predator detection, or general discouragement for predators to attack a larger‐sized group of individuals (Bshary & Noe, 1997; Chapman & Chapman, 2000; Oates & Whitesides, 1990). Individuals within mixed associations may also benefit from a combination of these advantages. For example, a decrease in vigilance behavior supported by mixed associations could provide better foraging efficiency (Podolsky, 1990), and increased predator detection may allow greater access to food resources in habitats that would otherwise be avoided (McGraw & Bshary, 2002).

Mixed species groups have been well documented in primates, including squirrel monkeys and capuchins in Brazil (Pinheiro et al., 2011), Goeldi's monkey and tamarins in Bolivia (Porter, 2001), langurs in Sri Lanka and Bangladesh (Al‐Razi et al., 2023; Lu et al., 2021), Diana and Campbell's monkeys in the Ivory Coast of Africa (Wolters & Zuberbühler, 2003), and macaques in China (Burton & Chan, 1996). However, none of the Indochina Colobinae primates have been observed to form mixed species group in the wild. Furthermore, mixed species primate groups are typically observed among closely related species (e.g., from members of the same genus), while mixed groups from distantly related species seem to be uncommon. Mixed species associations can range from relatively short encounters that last from hours or days (Terborgh, 1983) to stable groups that last from months or even years (Chapman & Chapman, 2000; Peres, 1992).

Here, we report new records of a mixed association between a group of Hatinh langur (*Trachypithecus hatinhensis*) and red‐shanked douc (*Pygathrix nemaeus*) from central Vietnam. The records are, to the best of our knowledge, the first documentation of a mixed association between these two primates in the wild.

## 2 MATERIALS AND METHODS

**FIGURE 1:**
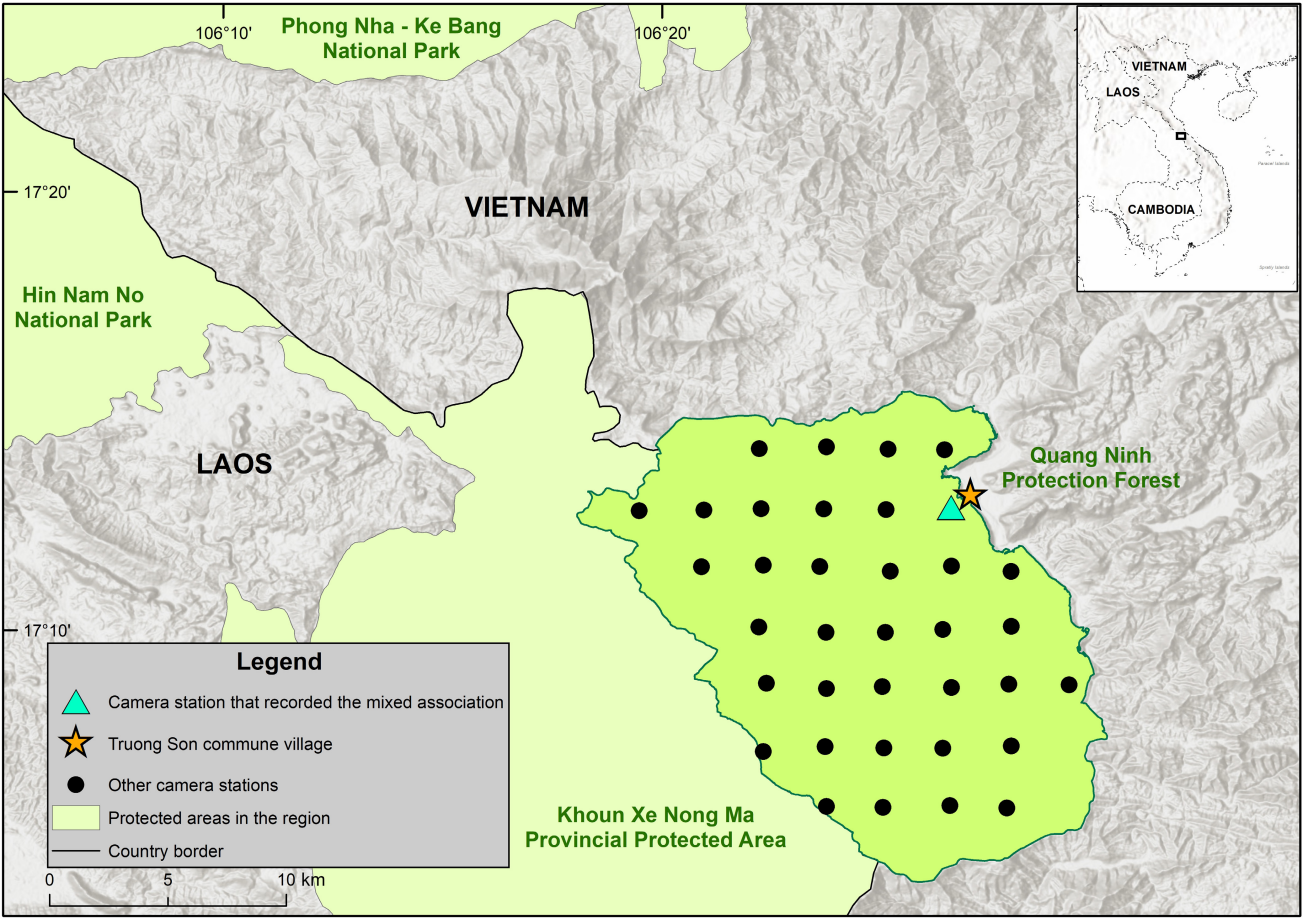
Camera‐trapping stations in Quang Ninh Protection Forest, Quang Binh, Vietnam.

From April 12, 2022 to August 24, 2022, we conducted a systematic camera‐trap survey, with overall protocols followed recommendations from Abrams et al. (2018), in Quang Ninh Protection Forest, located in Quang Binh Province, central Vietnam. In particular, camera stations were spaced approximately 2.5 km apart in a grid. Two independent white‐flash camera traps (Covert Illuminator) were set at each station, with cameras facing different directions, and were positioned along wildlife trails to maximize animal detections. We consider the cameras to be independent in the sense that, because they were facing different directions—often on different animal trails—these units did not always record the same wildlife events (Abrams et al., 2018). Cameras were set 20–40 cm above the ground and programmed to take a three‐photo burst when triggered. The cameras were deployed for an average of 109.9 ± 3.9 days. When processing the data, we treated the two cameras at the same location as a single station; in practice, this means that detections from both units were grouped together, and that photos of the same species at both cameras within a 1‐h time period were considered to be a single event for that species. A total of 36 camera stations were set up across the survey area, but cameras were stolen at eight stations, and thus we were only able to retrieve 28 stations (Figure 1). We used *camtrapR* (Niedballa et al., 2016) to process the data. A 60‐min interval was employed to define independent detections at any given station.

## 3 RESULTS AND DISCUSSION

One camera‐trap station recorded both Hatinh langur and red‐shanked douc within a single group on two separate occasions. The camera trap station was active for 113 days. In addition to the Hatinh langur and red‐shanked douc, the station recorded 268 independent detections of other wildlife, including the pig‐tailed macaque (*Macaca leonina*), stump‐tailed macaque (*M. arctoides*), ferret‐badger (*Melogale* spp.), Pallas' squirrel (*Callosciurus erythraeus*), red‐cheeked squirrel (*Dremomys rufigenis*), spotted linsang (*Prionodon pardicolor*), yellow‐bellied weasel (*Mustela kathiah*), and yellow‐throated marten (*Martes flavigula*).

**FIGURE 2:**
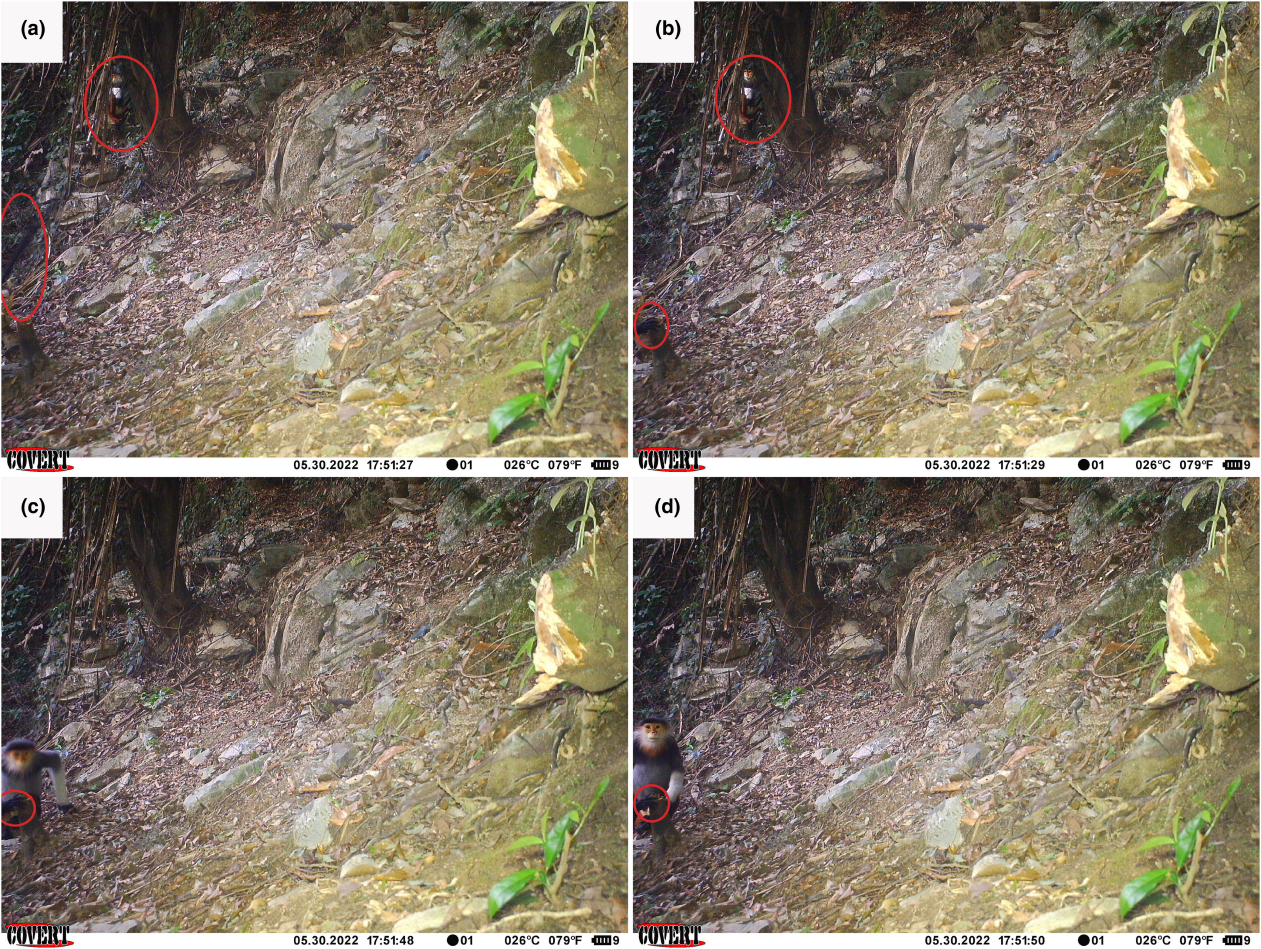
The first event showing a mixed association between Hathinh langur and red‐shanked douc. Note the characteristic black tail in (a), and black back hand of the Hatinh langur in (b–d).

The station was located near the top of a karst outcrop and close to the Truong Son commune village. The first event occurred on May 30, 2022. At 13:51, at least five Hatinh langurs passed through the camera's field of view. At 17:51, a Hatinh langur moved and sat just out of the camera's field of view, but one of its hands was still visible on a tree stump. A mature red‐shanked douc was seen at the same moment, and about 20 seconds later it sat close to the Hatinh langur. Both animals later moved out of the camera's field of view (Figure 2).

**FIGURE 3:**
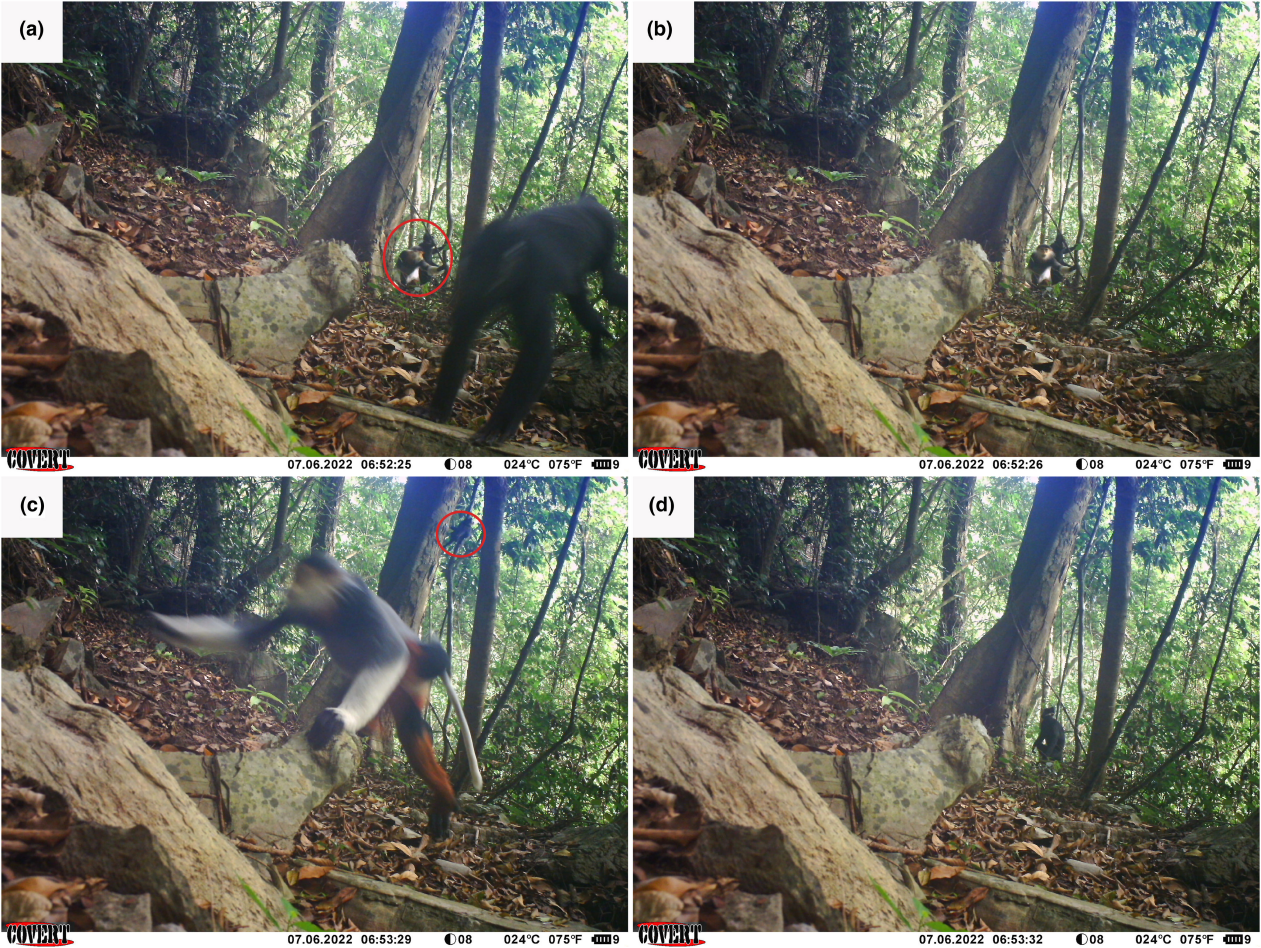
The second event showing a mixed association between Hatinh langurs and a red‐shanked douc. Note the distinct white tail and white back hand of the red‐shanked douc in panel (c). One Hatinh langur (the first langur) remained in the same position in all four photos in (a‐d).

The second event occurred on July 6, 2022, at the same station but on the other camera. At 06:52, two Hatinh langurs and one red‐shanked douc appeared in the camera's field of view. The red‐shanked douc sat close to one of the Hatinh langurs. The second Hatinh langur walked out of the frame, the red‐shank douc moved into the langur's position, and a third Hatinh langur started to enter the camera's field of view (panel c, Figure 3). The red‐shanked douc then left while the first langur stayed in the camera frame (Figure 3).

It is possible that both events show the same individuals, and therefore represent a semi‐stable group. Unfortunately, because we were unable to identify the primates to the individual level, we cannot say with certainty whether the two events showed the same animals. However, the fact that both events occurred at the same station, and both showed a single red‐shanked douc among multiple Hatinh langurs (Figures 2 and 3), provides some circumstantial evidence that both events show the same group of primates. If this is the case, then it is likely that the association would have been at least semi‐stable, as the two events were separated by 37 days.

This is, to the best of our knowledge, the first record of a mixed species association between Hatinh langur and red‐shanked douc that has been documented in the wild. Although there has been one report of interactions and subsequent hybridization between the two species, that event occurred in a primate rescue center in captive conditions, where the two species were kept in artificial proximity for an extended amount of time (Schempp et al., 2008), and therefore represents an unnatural occurrence.

There are several possible explanations for the mixed species association that we recorded. One possible explanation is that the formation of this interspecies group was facilitated by decreased populations of both Hatinh langur and red‐shanked douc in the Quang Ninh Protection Forest. Both taxa have declined precipitously in Vietnam in recent years through habitat loss and hunting (Coudrat et al., 2020; Quyet et al., 2021), and are becoming increasingly rare in Vietnam. Given the low visual encounter rates of primates compared to other sites in the region where our teams were conducting fieldwork, and the generally high level of hunting pressure in the area, it is likely that both primates are at highly depressed densities in the Quang Ninh Protection Forest. If the red‐shanked douc was unable to find other doucs in the area, it may have been compelled to join the Hatinh langur group.

Overall disturbance and degradation in the landscape may also have facilitated the mixed‐species association. Previous studies have shown increased mixed‐species associations in areas that are fragmented, comprised poor habitat quality, or have high levels of human disturbances (Lu et al., 2021). The camera trap station where we recorded the mixed‐species association was situated only 600 meters away from the main town of Truong Son Commune, in an area characterized by highly disturbed secondary and regenerating forests within a highly fragmented landscape. Human activity in the area was common. Therefore, although speculative, it is possible that the fragmented habitat, poor quality of remaining forests, and reduced dispersal potential to large areas of suitable habitat may have reduced the chances of both Hatinh langur and red‐shanked douc to meet their own species, and therefore form same‐species groups.

There could also be an ecological explanation for the association. Studies have shown that mixed‐species groups may help decrease predation risk (Bshary & Noe, 1997; Chapman & Chapman, 2000; Oates & Whitesides, 1990). We believe this is unlikely to be the case for the event that we observed, however, given that all large and medium‐sized mammalian carnivores—tiger *Panthera tigris*, leopard *Panthera pardus*, dhole *Cuon alpinus*, Asiatic golden cat *Catopuma temminckii*, and mainland clouded leopard *Neofelis nebulosa* – are likely extirpated in Vietnam (Goodrich et al., 2022; Gray et al., 2021; McCarthy et al., 2015; Stein et al., 2023), and are certainly absent from our study site. Mixed‐species groups have also been linked to improved foraging (Heymann & Buchanan‐Smith, 2000; Pinheiro et al., 2011; Porter, 2001). In some species, stable mixed‐species associations are associated with clearly differentiated feeding niches between species (Buchanan‐Smith, 1999). Because both Hatinh langur and red‐shanked douc are folivorous, with a presumably similar diet, we do not believe this is the case in the event that we observed. However, more detailed information on feeding preferences and foraging behavior of mixed associations between these species may give new insights into this topic.

One final possible explanation is that the red‐shanked douc represents an animal rescued from the wildlife trade and then released back to the wild. The illegal wildlife trade has been pervasive across Vietnam (Song, 2008), and included primates such as doucs (McEwan et al., 2021; Nadler & Roos, 2017). In many instances, authorities in Vietnam have released confiscated animals into the nearest forest, without proper considerations for best‐practice release protocols (Le et al., 2020; Nadler & Rosenthan, 1998). It is possible that the red‐shanked douc was reintroduced the Quang Ninh Protection Forest after being confiscated from the trade. If the animal was released into an area without other red‐shanked douc groups, then the single animal may have joined the Hatinh langur group as the next best alternative. Nonetheless, although this scenario is possible, we consider it implausible, as we were unaware of any rescue or release attempts of any doucs at the site through our exchanges with local authorities and rescue centers in the region.

Mixed associations are an important form of interspecies interactions among primates but remains little explored for most primate taxa, especially in Indochina. To our knowledge, our records represent the first report of a mixed association between the Hatinh langur and the red‐shanked douc that has been documented in the wild. By describing these events, we seek to lay a foundation for future studies to the potential drivers behind this behavior. Given the increase in camera trapping studies in Indochinese regions over the last decade, it is likely that similar mixed species groups will be recorded for other primates. We expect that observations over larger landscapes will allow for more in‐depth interpretations to be made on this interesting phenomenon.

## AUTHOR CONTRIBUTIONS

**Anh Tuan Nguyen:** Conceptualization (equal); formal analysis (lead); investigation (lead); writing – original draft (equal); writing – review and editing (equal). **Minh Le:** Formal analysis (supporting); investigation (supporting); writing – original draft (equal); writing – review and editing (equal). **Andrew Tilker:** Conceptualization (equal); formal analysis (supporting); writing – original draft (equal); writing – review and editing (equal).

## FUNDING INFORMATION

Nguyen Tuan Anh, ID VNU.2021.NCS.02, thanks The Development Foundation of Vietnam National University, Hanoi for sponsoring this research.

## CONFLICT OF INTEREST STATEMENT

The authors declare no conflict of interest.

## Data Availability

All datasets are included in the paper's main text.

## References

[ece370122-bib-0001] Abrams, J. F. , Axtner, J. , Bhagwat, T. , Mohd‐Azlan, J. , Nyugen, A. , Niedballa, J. , Sollmann, R. , Tilker, A. R. , & Wilting, A. (2018). Studying terrestrial mammals in tropical rainforests – a user guide for camera‐trapping and environmental DNA. Leibniz Institute for Zoo and Wildlife Research.

[ece370122-bib-0002] Al‐Razi, H. , Sattar, A. , Maria, M. , Guala, C. , & Nekaris, K. A. I. (2023). Mixed‐species association and a record of a hybrid offspring between *Trachypithecus pileatus* and *Trachypithecus phayrei* in Bangladesh. Primates, 64, 9–15. 10.1007/s10329-022-01035-8 36383280 PMC9842557

[ece370122-bib-0003] Bshary, R. , & Noe, R. (1997). Red colobus and Diana monkeys provide mutual protection against predators. Animal Behaviour, 54, 1461–1474. 10.1006/anbe.1997.0553 9794772

[ece370122-bib-0004] Buchanan‐Smith, H. M. (1999). Tamarin polyspecific associations: Forest utilization and stability of mixed‐species groups. Primates, 40, 233–247. 10.1007/BF02557713 23179543

[ece370122-bib-0005] Burton, F. D. , & Chan, L. (1996). Behavior of mixed species groups of macaques. In J. E. Fa & D. G. Lindburg (Eds.), Evolution and ecology of macaque societies. Cambridge University Press.

[ece370122-bib-0006] Chapman, C. A. , & Chapman, L. J. (2000). Interdemic variation in mixed‐species association patterns: Common diurnal primates of Kibale National Park, Uganda. Behavioral Ecology and Sociobiology, 47, 129–139. 10.1007/s002650050003

[ece370122-bib-0007] Coudrat, C. N. Z. , Quyet, L. K. , Duc, H. , Phiaphalath, P. , Rawson, B. M. , Nadler, T. , Ulibarri, L. , & Duckworth, J. W. (2020). Pygathrix nemaeus, red‐shanked douc. The IUCN Red List of Threatened Species.

[ece370122-bib-0008] Goodale, E. , Sridhar, H. , Sieving, K. E. , Bangal, P. , Colorado, Z. G. J. , Farine, D. R. , Heymann, E. W. , Jones, H. H. , Krams, I. , Martínez, A. E. , Montaño‐Centellas, F. , Muñoz, J. , Srinivasan, U. , Theo, A. , & Shanker, K. (2020). Mixed company: A framework for understanding the composition and organization of mixed‐species animal groups. Biological Reviews, 95, 889–910. 10.1111/brv.12591 32097520 PMC7383667

[ece370122-bib-0009] Goodrich, J. , Wibisono, H. , Miquelle, D. , Lynam, A. J. , Sanderson, E. , Chapman, S. , Gray, T. N. E. , Chanchani, P. , & Harihar, A. (2022). Panthera tigris, tiger. The IUCN Red List of Threatened Species.

[ece370122-bib-0010] Gray, T. , Borah, J. , Coudrat, C. N. Z. , Ghimirey, Y. , Giordano, A. , Greenspan, E. , Petersen, W. , Rostro‐García, S. , Shariff, M. , & Wai‐Ming, W. (2021). Neofelis nebulosa, clouded leopard. The IUCN Red List of Threatened Species.

[ece370122-bib-0011] Haugaasen, T. , & Peres, C. A. (2009). Interspecific primate associations in Amazonian flooded and unflooded forests. Primates, 50, 239–251. 10.1007/s10329-009-0135-4 19242777

[ece370122-bib-0012] Heymann, E. W. , & Buchanan‐Smith, H. M. (2000). The behavioural ecology of mixed‐species troops of callitrichine primates. Biological Reviews, 75, 169–190. 10.1111/j.1469-185X.1999.tb00044.x 10881387

[ece370122-bib-0013] Le, M. D. , McCormack, T. E. M. , Hoang, H. V. , Duong, H. T. , Nguyen, T. Q. , Ziegler, T. , Nguyen, H. D. , & Ngo, H. T. (2020). Threats from wildlife trade: The importance of genetic data in safeguarding the endangered four‐eyed turtle (*Sacalia quadriocellata*). Nature Conservation, 41, 91–111. 10.3897/natureconservation.41.54661

[ece370122-bib-0014] Lu, A. , Sirimanna, D. G. R. , Wijayathunga, L. , Vandercone, R. , & Salmi, R. (2021). Mixed‐species associations and attempted mating suggest hybridization between purple‐faced and tufted gray langurs of Sri Lanka. Primates, 62, 11–17. 10.1007/s10329-020-00852-z 32804328 PMC7430210

[ece370122-bib-0015] McCarthy, J. , Dahal, S. , Dhendup, T. , Gray, T. N. E. , Mukherjee, S. , Rahman, H. , Riordan, P. , Boontua, N. , & Wilcox, D. (2015). Catopuma temminckii, Asiatic golden cat. The IUCN Red List of Threatened Species.

[ece370122-bib-0016] McEwan, A. , Nadler, T. , & Nevin, O. (2021). The illegal trade of the douc langurs (*Pygathrix sp*.) in Vietnam – January 2010 to December 2020. Vietnamese Journal of Primatology, 3, 157–170.

[ece370122-bib-0017] McGraw, W. S. , & Bshary, R. (2002). Association of terrestrial mangabeys (*Cercocebus atys*) with arboreal monkeys: Experimental evidence for the effects of reduced ground predator pressure on habitat use. International Journal of Primatology, 23, 311–325. 10.1023/A:1013883528244

[ece370122-bib-0018] Nadler, T. , & Roos, C. (2017). Impending extinction crisis of the world's primates – implications for Vietnam. Vietnamese Journal of Primatology, 2, 25–35.

[ece370122-bib-0019] Nadler, T. , & Rosenthan, S. (1998). Wildlife rescue centers and problems of confiscated wild animals in Vietnam. Zoo's Print, 13, 5–8.

[ece370122-bib-0020] Niedballa, J. , Sollmann, R. , Courtiol, A. , & Wilting, A. (2016). camtrapR: An R package for efficient camera trap data management. Methods in Ecology and Evolution, 7, 1457–1462. 10.1111/2041-210X.12600

[ece370122-bib-0021] Oates, J. F. , & Whitesides, G. H. (1990). Association between olive colobus (*Procolobus verus*), Diana guenons (*Cercopithecus diana*), and other forest monkeys in Sierra Leone. American Journal of Primatology, 21, 129–146. 10.1002/ajp.1350210206 31963982

[ece370122-bib-0022] Peres, C. A. (1992). Prey‐capture benefits in a mixed‐species group of Amazonian tamarins, *Saguinus fuscicollis* and *S. Mystax* . Behavioral Ecology and Sociobiology, 31, 339–347. 10.1007/BF00177774

[ece370122-bib-0023] Pinheiro, T. , Ferrari, S. F. , & Lopes, M. A. (2011). Polyspecific associations between squirrel monkeys (*Saimiri sciureus*) and other primates in eastern Amazonia. American Journal of Primatology, 73, 1145–1151. 10.1002/ajp.20981 21809365

[ece370122-bib-0024] Podolsky, R. D. (1990). Effects of mixed‐species association on resource use by *Saimiri sciureus* and *Cebus apella* . American Journal of Primatology, 21, 147–158. 10.1002/ajp.1350210207 31963980

[ece370122-bib-0025] Porter, L. M. (2001). Benefits of polyspecific associations for the Goeldis monkey (*Callimico goeldii*). American Journal of Primatology, 54, 143–158. 10.1002/ajp.1019 11443630

[ece370122-bib-0026] Quyet, L. K. , Coudrat, C. N. Z. , Phiaphalath, P. , Nadler, T. , & Covert, H. (2021). Trachypithecus hatinhensis, Hatinh langur. The IUCN Red List of Threatened Species.

[ece370122-bib-0027] Schempp, W. , Munch, C. , Roos, C. , & Nadler, T. (2008). Chromosomal and molecular studies of a hybrid between red‐shanked douc langur (*Pygathrix nemaeus*) and Hatinh langur (*Trachypithecus laotum hatinhensis*). Vietnamese Journal of Primatology, 2, 55–62.

[ece370122-bib-0028] Song, N. V. (2008). Wildlife trading in Vietnam: Situation, causes, and solutions. Journal of Environment & Development, 17, 145–165. 10.1177/1070496508316220

[ece370122-bib-0029] Stein, A. B. , Athreya, V. , Gerngross, P. , Balme, G. , Henschel, P. , Karanth, U. , Miquelle, D. , Rostro‐García, S. , Kamler, J. F. , Laguardia, A. , Khorozyan, I. , & Ghoddousi, A. (2023). Panthera pardus, leopard. The IUCN Red List of Threatened Species.

[ece370122-bib-0030] Terborgh, J. (1983). Five new world primates: A study in comparative ecology. Princeton University Press.

[ece370122-bib-0031] Terborgh, J. (1990). Mixed flocks and polyspecific associations: Costs and benefits of mixed groups to birds and monkeys. American Journal of Primatology, 21, 87–100. 31963979 10.1002/ajp.1350210203

[ece370122-bib-0033] Wolters, S. , & Zuberbühler, K. (2003). Mixed‐species associations of Diana and Campbell's monkeys: The costs and benefits of a forest phenomenon. Behaviour, 140, 371–385. 10.1163/156853903321826684

